# Identifying drugs with the greatest increases and decreases in spending per beneficiary using Medicare Part D: A cross-sectional study

**DOI:** 10.1371/journal.pone.0281076

**Published:** 2023-02-09

**Authors:** Benazir Hodzic-Santor, Chana A. Sacks, Tamara Van Bakel, Michael Fralick

**Affiliations:** 1 Division of General Internal Medicine, Department of Medicine, Sinai Health System, Toronto, Ontario, Canada; 2 Division of General Internal Medicine and Mongan Institute, Department of Medicine, Massachusetts General Hospital, Harvard Medical School, Boston, MA, United States of America; 3 Lunenfeld-Tanenbaum Research Institute, Sinai Health System, Toronto, Ontario, Canada; The University of Texas MD Anderson Cancer Center, UNITED STATES

## Abstract

**Importance:**

In the US, there are no effective regulations controlling how much the price of a medication can increase. A patchwork of studies examining the reasons for soaring prices has focused on medications that have received considerable media attention, like insulin, epinephrine, and colchicine.

**Objective:**

To identify the 50 medications with the greatest increase in average spending per beneficiary and the 50 medications with the greatest decrease in average spending per beneficiary, and to identify the factors associated with spending increases.

**Design, participants:**

This cross-sectional study used publicly available data from the Medicare Part D Prescription Drug Program from 2014 to 2020. We included drugs dispensed to > 1000 beneficiaries in each study year and excluded those primarily administered intravenously.

**Main measures:**

Percentage change in average spending per beneficiary from 2014 to 2020 was calculated for each drug. For each drug, we extracted the number of beneficiaries, the number of manufacturers, and the drug-specific total annual spending reported in the Medicare Part D data set. An online database search was conducted to identify the primary clinical indication, the availability of any generic versions, and the date of FDA approval for each drug.

**Results:**

The 50 medications with the greatest increase in spending per beneficiary had a median increase of 362.4% (interquartile range [IQR]: 286.6%-563.0%), with a cumulative spending of almost $5 billion in 2020 alone. Most drugs with the greatest increases in spending per beneficiary had generic versions available (68%) and were approved by the FDA over 10 years ago (66%). Medications with the greatest increase in spending per beneficiary had a median of 1 manufacturer (IQR: 1–2), while medications with the greatest decrease in spending per beneficiary had a median of 9.5 manufacturers (IQR: 5–14).

**Conclusions:**

This study identified rapidly increasing costs of medications under Medicare Part D. Our findings demonstrate that off-patent medications can skyrocket in price, especially when there are few manufacturers of a given medication.

## Introduction

The United States spends more per capita on prescription medications than any other country in the world [1]. Several interconnected factors drive this reality. First, until very recently, there have been no limits or effective regulations determining how high a medication can be priced once it reaches the market, or how much the price can be increased each year thereafter. This has resulted in medications, including medications that have been on the market for many decades, experiencing exponential rises in price. Salient examples that have received considerable attention in both the lay and scientific press include insulin [2], epinephrine [3], and colchicine [4]. Second, Food and Drug Administration (FDA) approval provides market exclusivity until patent expiration, which severely limits competition for an identical molecule [1].

A patchwork of literature identifies some of the reasons why certain off-patent generic medications have skyrocketed in price [5, 6]. The reasons span from outright securities fraud [7] to drug shortages, and a unifying theme is a lack of competition. However, there has been little systematic research to identify whether such explanations pertain only to specific examples that have received media attention. Furthermore, without an available comparator to medications not experiencing marked rises in price, it is challenging to empirically identify reasons for the price rises. The objective of our study was to identify the 50 medications with the greatest increase in average spending per beneficiary and the 50 medications with the greatest decrease in average spending per beneficiary between 2014 and 2020, and to compare the characteristics of these medications to identify factors associated with spending increases.

## Methods

### Data set

We conducted a cross-sectional study using publicly available data from the Medicare Part D Prescription Drug Program (2014 to 2020) [3, 4]. Specifically, we used the publicly available data on the Medicare Part D Dashboard (https://data.cms.gov/summary-statistics-on-use-and-payments/medicare-medicaid-spending-by-drug/medicare-part-d-spending-by-drug). The Medicare Part D data set was developed by the Centers for Medicare & Medicaid Services (CMS) and includes information for Medicare beneficiaries who are enrolled in Part D, which represents about 70% of all Medicare beneficiaries [3, 4]. The Medicare Part D Prescription Drug Program data is publicly available, and thus institutional review board approval was not required.

### Cohort creation

We first excluded all medications that were dispensed to 1000 beneficiaries or less in any of the years between 2014 and 2020. This was done to exclude drugs for rare conditions that affect a small patient population, as well as drugs that went off the market during this time. We then rank-ordered the drugs based on percentage increase or decrease in average spending per beneficiary from 2014 to 2020, identifying the 50 drugs with the greatest increase, as well as the 50 drugs with the greatest decrease. Drugs that were primarily administered intravenously were excluded, as the Part D data set has incomplete capture of these medications.

### Exposure

Our exposure of interest was the percentage change in average spending per beneficiary over the study time period.

### Variables

The Medicare Part D data set includes drug spending metrics for both brand name and generic medications; these metrics are based on the gross drug cost, including ingredient cost, dispensing fees, and sales tax. In this data set, total spending includes amounts paid by the Medicare Part D plan, as well as beneficiary payments, which vary depending on decisions made at the level of each Part D plan administration. Pricing information is reported separately for brand name and generic versions of the same medication.

For each medication in the Medicare Part D data set, we extracted the generic drug name, the brand name (where applicable), the number of beneficiaries prescribed each drug, the number of claims made, the drug-specific total annual spending reported by Medicare, and the average spending per year per beneficiary. The Medicare data set also provides manufacturer information for each drug: from these data, we extracted the number of manufacturers for each drug and the name and number of manufacturers by year. A search of the Lexicomp Online drug information database was used to identify the primary clinical indications for each drug, as well as the availability of any generic versions. A search of the Orange Book Database was conducted to identify the year of FDA approval and availability of any generic versions.

### Statistical analysis

We compared the baseline characteristics of the 50 drugs with the greatest increase in spending per beneficiary to the baseline characteristics of the 50 drugs with the greatest decrease in spending per beneficiary. Chi-square test was used for binary variables and student’s T test for continuous variables. We plotted the number of beneficiaries per year against the change in average spending per beneficiary. Finally, we examined how the average spending per beneficiary varied by manufacturer. Percentage change was calculated by taking the difference in average spending per beneficiary from 2014 to 2020 and dividing by the average spending per beneficiary in 2014. All analyses were performed using the ‘stats’ package in R Statistical Software (version 4.0.5; R Foundation for Statistical Computing, Vienna, Austria).

## Results

**Fig 1:**
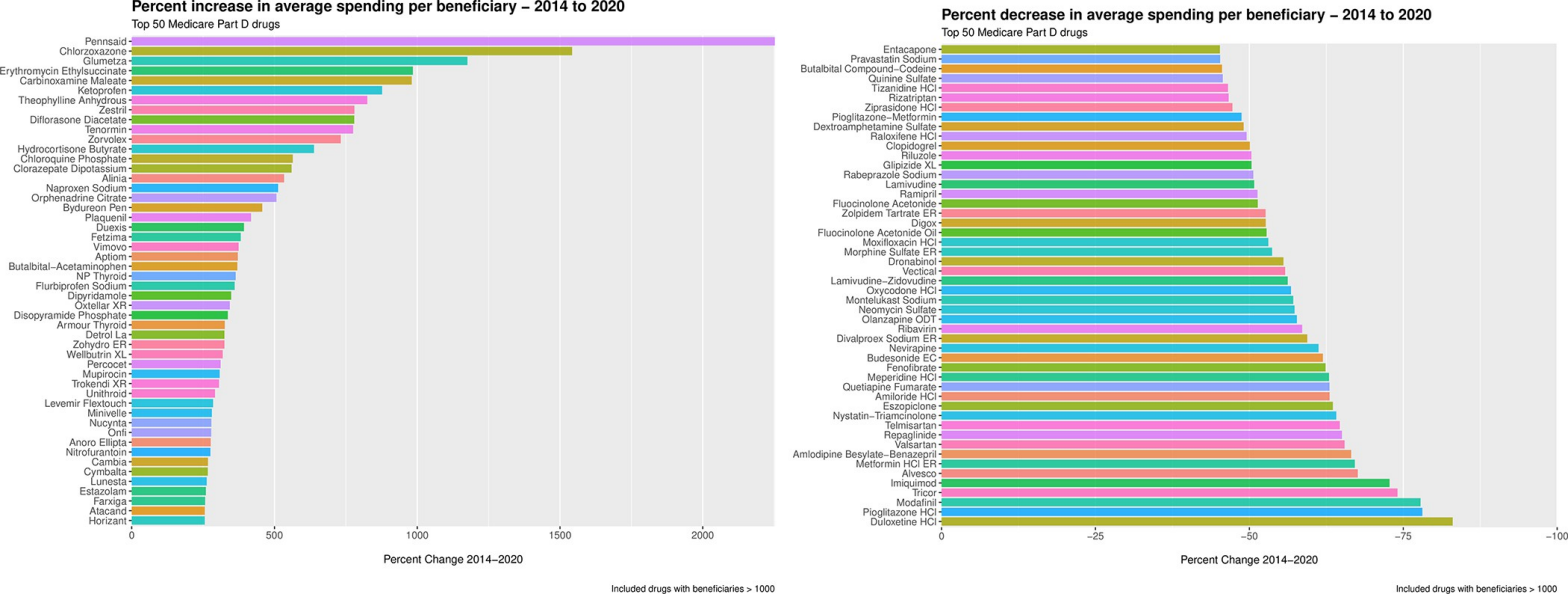
Top 50 drugs with (A) greatest percentage increase and (B) greatest percentage decrease in average spending per beneficiary between 2014 and 2020.

**Fig 2:**
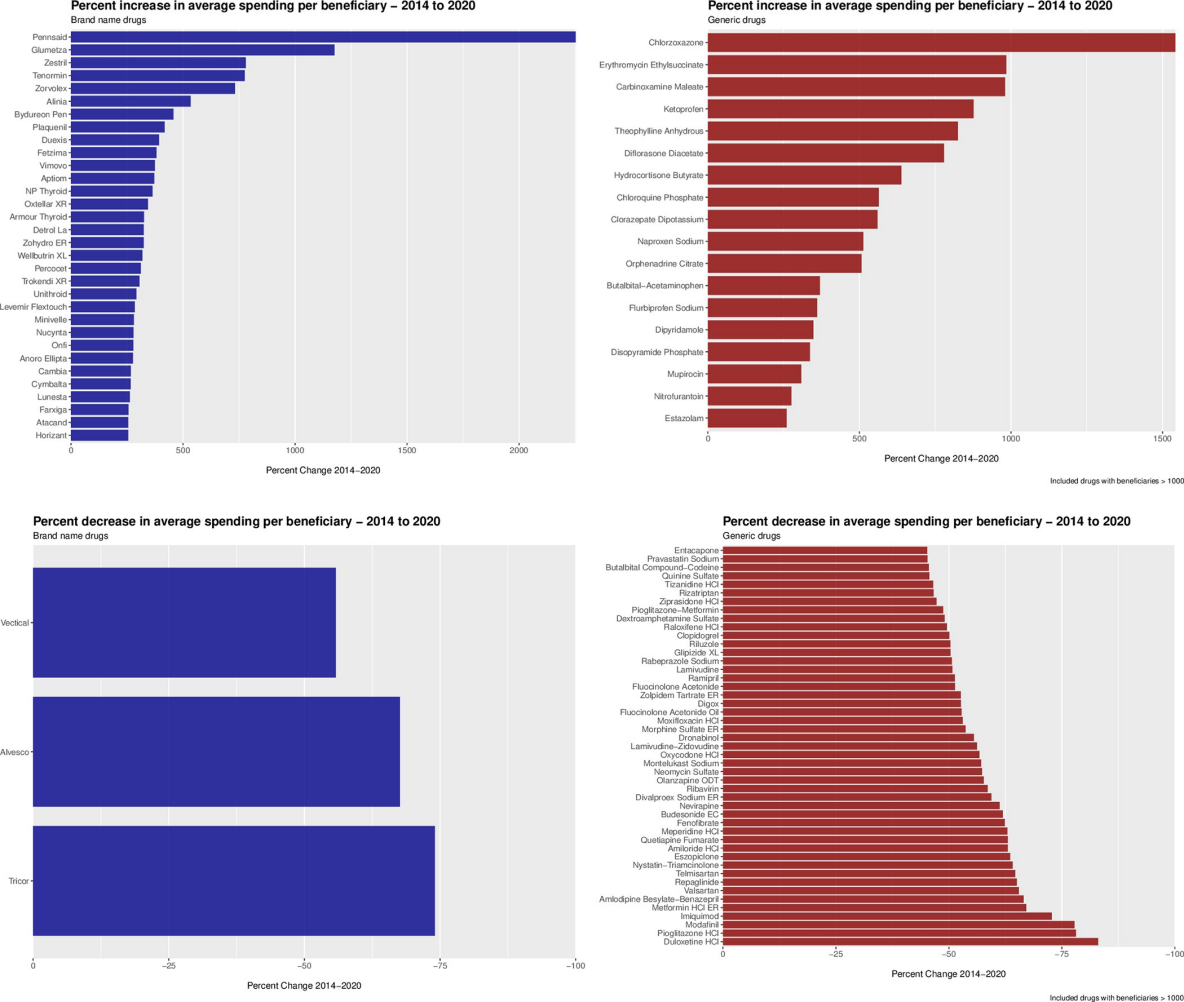
Percentage (A) increase or (B) decrease in average spending per beneficiary between 2014 and 2020 categorized by brand-name and generic drugs.

After excluding drugs with 1000 beneficiaries or less, we were left with 1066 drugs. The 50 drugs with the greatest percentage increase in average spending per beneficiary are displayed in Fig 1A. (For percentage change visualized separately for brand-name and generic drugs, see Fig 2A.).

**Fig 3:**
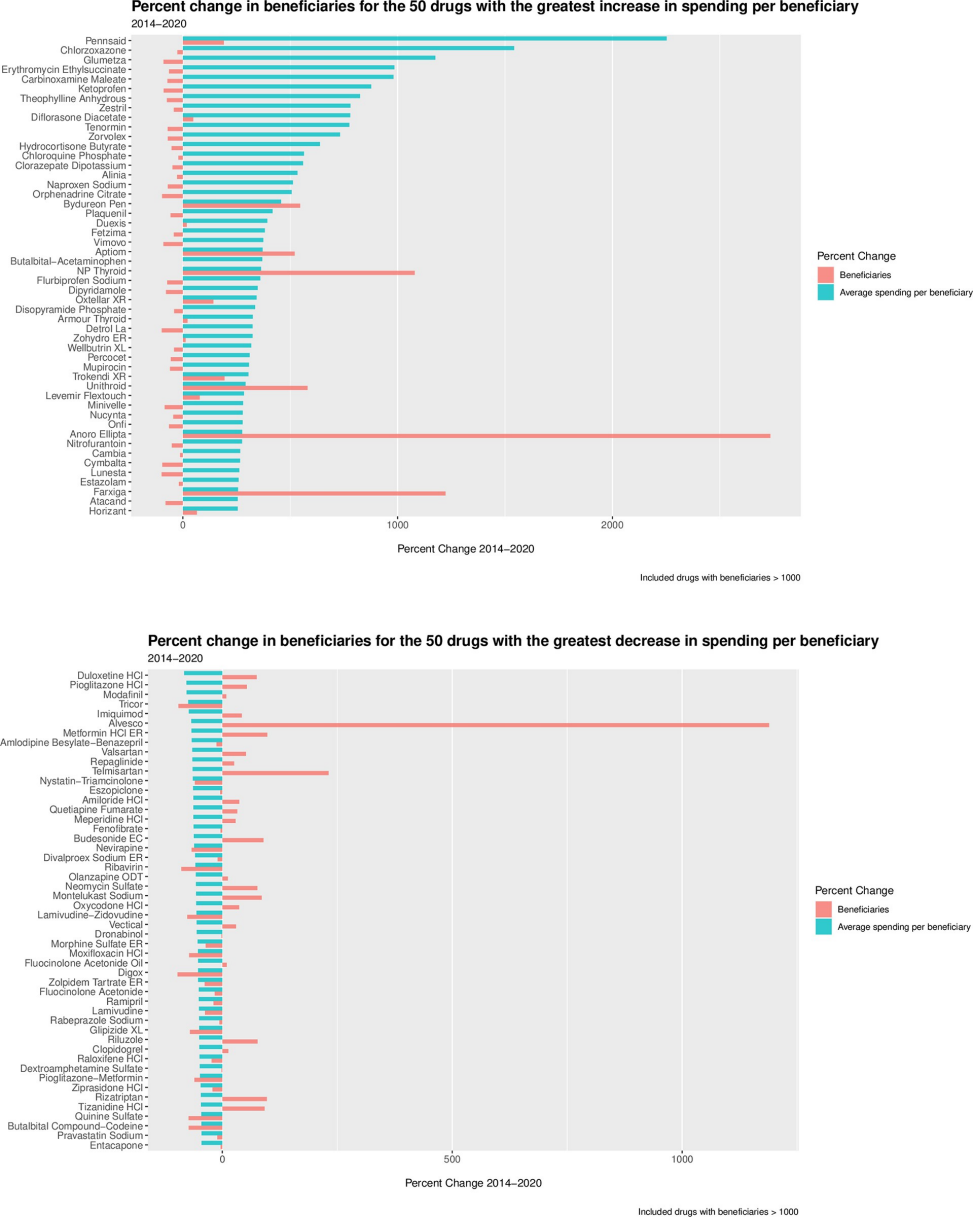
Percentage change in beneficiaries for the top 50 drugs with (A) greatest increase and (B) greatest decrease in average spending per beneficiary between 2014 and 2020.

Across the 50 drugs with the greatest increases in spending, the median average spending per beneficiary was $574.76 (IQR: 113.61–1063.40) in 2014, jumping to $3,269.36 (IQR: 801.60–4655.31) in 2020, corresponding to a 468.8% increase in spending over the study period and a 362.4% median increase. Of these 50 drugs, 3 had increases in average spending per beneficiary between 1000% and 2253%, 14 had spending increases between 500% and 1000%, and the remaining 33 drugs had spending increases between 255% and 500%. The median number of beneficiaries, per drug, was 6733 (IQR: 4001–24,956) in 2014 and 5038 (IQR: 1758–13,962) in 2020. The total number of beneficiaries across all 50 drugs was 1,487,430 in 2014 and 1,522,953 in 2020. (Percentage change in beneficiaries is featured in Fig 3A.) The median number of claims, per drug, was 28,545 (IQR: 9025–84,012) in 2014 and 21,192 (IQR: 6420–72,817) in 2020. Across these 50 drugs, total spending in was $871,084,865 in 2014 and $4,640,971,268 in 2020. Overall, spending on these 50 drugs accounts for 2.3% of the $198 billion estimated Part D spending in 2020.

The 50 drugs with the greatest increases in spending per beneficiary were used to treat a variety of conditions: 13 (26%) were used for pain management, 12 (24%) were used to treat neurological or psychiatric conditions, 8 (16%) were used to treat endocrine conditions, 5 (10%) were used to treat cardiovascular conditions, 5 (10%) were used to treat infectious disease, 4 (8%) were used to treat allergy/immunological conditions, 2 (4%) were used to treat respiratory conditions, and 1 (2%) drug (i.e., Detrol) did not fall into any of these categories (Table 1).

**Table 1:** Characteristics of the included medications.

	Top 50 Increase in spending per beneficiary	Top 50 Decrease in spending per beneficiary
**Drug category**		
Cardiovascular	5 (10%)	10 (20%)
Endocrinology	8 (16%)	6 (12%)
Respirology	2 (4%)	2 (4%)
Neuro/Psychiatric	12 (24%)	13 (26%)
Gastroenterology	0	1 (2%)
Infectious diseases	5 (10%)	7 (14%)
Pain	13 (26%)	4 (8%)
Allergy and immunology	4 (8%)	4 (8%)
Vitamin/Electrolyte	0	1 (2%)
Other	1 (2%)	2 (4%)
**Brand name only available**	16 (32%)	1 (2%)
**Number of manufacturers (median)**	1 (IQR: 1–2)	9.5 (IQR: 5–14)

The 50 drugs with the greatest percentage decrease in average spending per beneficiary are displayed in Fig 1B. (For percent change visualized separately for brand-name and generic drugs, see Fig 2B.) Among these 50 drugs, the median average spending per beneficiary was $546.95 (IQR: 293.88–1113.13) in 2014, falling to $215.36 (IQR: 90.6–484.58) in 2020, corresponding to a 60.6% decrease in spending over the study period. The median number of beneficiaries, per drug, was 70,983 (IQR: 25,685–445,363) in 2014 and 82,209 (IQR: 18,265–376,534) in 2020. (Percentage change in beneficiaries is featured in Fig 3B.) The median number of claims, per drug, was 265,931 (IQR: 71,987–2,282,972) in 2014 and 226,870 (IQR: 52,804–1,778,776) in 2020. Across these 50 drugs, total spending was $6,434,197,482 in 2014 and $2,730,896,942 in 2020.

The 50 drugs with the greatest decrease in spending per beneficiary were used to treat a variety of conditions: 13 (26%) were used to treat neurological or psychiatric conditions, 10 (20%) were used to treat cardiovascular conditions, 7 (14%) were used to treat infectious disease, 6 (12%) were used to treat endocrine conditions, 4 (8%) were used to treat allergy/immunological condition, 4 (8%) were used for pain management, 2 (4%) were used to treat respiratory conditions, 1 (2%) was used to treat gastroenterological conditions, 1 (2%) was classified as a vitamin/electrolyte, and 2 (4%) of the drugs (i.e., Dronabinol and quinine sulfate) did not fall into any of these categories (Table 1).

Across the 50 drugs with the greatest increases in spending per beneficiary, the median number of manufacturers over the course of the study period was 1 (IQR: 1–2), while in the group with the greatest decreases in spending, the median number of manufacturers was 9.5 (IQR: 5–14, *p* = 0.009).

Of the drugs with the greatest increases in spending per beneficiary, 48 were included in Orange Book, and the majority (i.e., 66%) were approved more than 10 years prior to the start of the study period. Specifically, 11 were approved within 5 years of the study start date (2014), 4 were approved in the preceding 5–10 years, 7 were approved in the preceding 10–20 years, and 26 were approved over 20 years before 2014. Of the 50 drugs with the greatest increase in spending per beneficiary, 32 (64%) were brand name and 16 (32%) did not have a generic available from 2014 to 2020.

Of the drugs with the greatest decrease in spending per beneficiary, 5 were approved in the preceding 5–10 years, 29 were approved in the preceding 10–20 years, and 16 were approved over 20 years before 2014. All but one of the drugs (i.e., Alvesco) with the greatest spending decrease had generic versions available from 2014 to 2020. Of these 50 drugs, 3 (6%) were brand name.

## Discussion

In this nationwide study of medications from the Medicare Part D Prescription Drug Program from 2014 to 2020, the 50 medications with the greatest increase in spending per beneficiary had a median increase of 362.4%, with a cumulative reported spending of almost $5 billion in 2020 alone. For both the 50 medications with the greatest spending increases and the 50 medications with the greatest spending decreases, generic alternatives were available for most medications, but the number of available manufacturers over the course of the study period was 9-fold higher for the medications with the greatest spending decreases. Furthermore, for both groups of medications, the majority were FDA-approved in 2004 or earlier. These findings demonstrate that off-patent medications can skyrocket in price.

A common reason why medications may increase in price is a lack of competition. A recent FDA report analyzing drugs sold to retail pharmacies that had their initial generic entry into the US market between 2015 and 2017 showed the association between percentage reductions in average manufacturer price (AMP) and number of generic competitors as follows: with 1 generic producer, there was a 39% reduction in price from brand AMP (prior to generic competition) to generic AMP; with 2 generic producers, a 54% reduction in price; with 4 generic producers, a 79% reduction in price; and with 6 or more generic competitors on the market, a drug’s AMP underwent reductions of over 95% [8]. Other research has supported the association, noting that the rate of price reduction generally decreases for each additional manufacturer [9, 10]. Our study also supports this association, given that the 50 medications with the greatest increase in spending per beneficiary had, on average, only 1 manufacturer, compared to the average 9.5 manufacturers for the 50 medications with the greatest decreases in spending.

In addition to limiting competition, a low number of manufacturers can also create vulnerable markets in the event of a drug shortage. Among the 50 medications with the greatest increases in average spending per beneficiary, several medications experienced a drug shortage during our study period, including bupropion (319% increase in spending), theophylline (825% increase in spending), and disopyramide phosphate (337% increase in spending) [11–14]. The causes of drug shortages can include disruptions in manufacturing due to failures in quality management, issues procuring raw materials, and natural disasters [15]. While the FDA maintains a list of drugs currently in shortage, it remains challenging to definitively identify which drugs experienced a shortage during the study period because there is no publicly accessible database featuring historical data on drug shortages.

Prior work has identified other reasons why drugs increase in price, such as the marketing of combination pills that combine inexpensive generic constituents into a new, branded, fixed-dose combination product [6, 16]. Among the drugs with the greatest price increases, Duexis (a combination of ibuprofen and famotidine) and Vimovo (naproxen and esomeprazole) are illustrative examples [16]. We also observed that most (66%) of the drugs with the greatest increase in spending per beneficiary had been approved more than 10 years before the start of the study period, and that most (68%) had generic versions available from 2014 to 2020. This observation conflicts with the conventional wisdom that drugs with the greatest increases in price are “new,” and that the introduction of generic medications drives down prices by increasing manufacturer competition [17]. Although drug prices may drop after the introduction of a generic, generic drug industry consolidation, mergers, and discontinuation of products due to thin profit margins have resulted in waning competition over time [18]. Furthermore, brand-name manufacturers may prevent or delay competition through the application of secondary and tertiary patents to extend brand name exclusivity [17, 19, 20]. Indeed, it is estimated that the majority of generic drugs are manufactured by only one or two companies, with 40% of generic drugs produced by only a single manufacturer [17].

It is important to contextualize our findings within the total spending on medications covered under Part D. In recent years, Medicare has spent over $160 billion per year on Part D medications. Although the 50 drugs with the highest increases in spending per beneficiary make up a small proportion of this total spending, from the patient’s perspective, unpredictability in drug spending that results from year-to-year changes in the prices of their medications is critically important and potentially highly disruptive. The Inflation Reduction Act (IRA), which was signed into law in August 2022, has provisions that may prevent medications from skyrocketing in price in the future [21]. Beginning in October 2022, the IRA requires that drug companies pay rebates to Medicare if prices for drugs increase faster than the rate of inflation; this provision will apply to nearly all drugs covered under Part D. It is anticipated this measure will reduce out-of-pocket drug spending for Medicare beneficiaries, slow year-over-year price increases, and result in a net federal deficit reduction of $62 billion between the years of 2022 and 2031 (including $71 billion in savings to Medicare, partially offset by additional Medicaid spending) [22]. Further, through its Drug Price Negotiation Program (starting in 2026), the IRA enables the Secretary of Health & Human Services to negotiate with manufacturers on the price of a select number of the costliest single-source brand-name drugs without generic competitors that are covered under Medicare Part D. These provisions of the IPA are substantial and transformative, though not airtight. For instance, there are no mechanisms in the IRA to lower or control launch prices; consequently, companies may be incentivized to increase their launch prices to offset inflation-based pricing restrictions. The impact of the IRA on drug prices will be a critical area for future study.

### Limitations

There are a number of limitations to our study. First, Medicare Part D predominantly includes adults over 65 years of age, and thus it is unknown whether these results also apply to medications prescribed to patients under age 65. Second, the precise reason why the average spending per beneficiary for any given drug increased or decreased over the course of the study period is unknowable, and thus we speculated based on the available literature and circumstances surrounding certain medications. Third, we may have overestimated the spending increases for the included brand-name medications because we lacked data on rebates. However, brand name drugs made up a small subset of the 50 medications with the greatest spending increases, and assuming the rebates are a fixed 40% each year [23], this doesn’t have a meaningful effect on the change in spending we’ve calculated. Furthermore, this potential limitation of overestimating price increases is counterbalanced by the fact the CMS data set only includes the 70% of Medicare beneficiaries who are enrolled in Part D.

## Conclusions

To understand the factors associated with rising prescription medication costs, we identified the 50 medications with the greatest increase in average spending per beneficiary and the 50 medications with the greatest decrease in average spending per beneficiary between 2014 and 2020. Our results demonstrated staggering spending increases, with the 50 medications with the greatest increases showing a median increase of 362.4%. Most of drugs with the greatest increases in spending had generic versions available and were approved by the FDA over 10 years prior to the start of the study period, yet on average had few manufacturers. Together with the existing literature, our results point towards the need for policy interventions to regulate drug prices in the US.
